# Calculation method of HJC constitutive model parameters of natural joint angle slate

**DOI:** 10.1038/s41598-023-42544-w

**Published:** 2023-09-14

**Authors:** Antong Wan, Tiejun Tao, Xingchao Tian, Caijin Xie, Jian Jia

**Affiliations:** 1 College of Civil Engineering, Guizhou University, Guiyang, 550025 China; 2 College of Mining, Guizhou University, Guiyang, 550025 China

**Keywords:** Civil engineering, Petrology

## Abstract

In the course of underground engineering, layered slate is often encountered. Understanding the mechanical characteristics of layered slate is a prerequisite for engineering construction and disaster prevention and mitigation. As a result, at the beginning of a project, a large number of indoor tests are required, which are time-consuming and laborious. In addition, the natural joint angle of the layered slate is random, so it is extremely difficult to establish a database of the mechanical characteristics of layered slate. Hence, it is necessary to find a simple, feasible, and high-precision method to determine the Holmquist–Johnson–Cook (HJC) constitutive model parameters for naturally jointed layered slate with different dip angles. This study first determines the HJC constitutive model parameters of layered slate with five specific joint dip angles (0°, 30°, 45°, 60°, and 90°) through static tests and the split Hopkinson pressure bar (SHPB) test. Furthermore, by employing sensitivity analysis methods, the influence of key parameters of the HJC constitutive model on the dynamic peak stress of slate is determined. Among them, parameters A and B have the most significant impact on stress, exceeding 50%. Thirdly, a nonlinear fitting regression method is used to determine the HJC constitutive model parameters of naturally jointed angular slate. The relationship between the HJC model parameters and the inclination angle of slate joints is derived, and the accuracy of these parameters is verified through numerical simulation methods. The error between the numerical simulation and indoor experiments is within 10%, indicating a high level of simulation accuracy. The research findings provide a highly precise numerical simulation method for similar projects.

## Introduction

Slate is a common rock mass encountered in geotechnical engineering in China. It is of great significance to study the dynamic characteristics of slate under impact loading for accurate rock-breaking and disaster prevention and mitigation in geotechnical engineering^1–3^. Numerical simulation analysis is a common method used to solve slate dynamics problems, and the selection of the constitutive model and the determination of parameters are the keys to such simulation, directly affecting the validity and accuracy of the simulation results^4^.

Some scholars have incorporated the Holmquist–Johnson–Cook (HJC) constitutive model into the numerical simulation analysis of rock dynamics and have performed a great deal of research on the parameter value method. Fang et al.^5^ proposed a method to determine the parameters of the HJC constitutive model of Salem limestone and extended it to general rock materials. Li et al.^6^ conducted numerical simulations of coal rock SHPB experiments based on the HJC constitutive model and the finite element software LS-DYNA. The results of the study showed that the damage to coal rock was affected by both compression and tension when the impact velocity was 3 m/s, and when the impact velocity was 9 m/s, the damage to coal rock was mainly affected by impact compression. Shi et al.^7^. constructed a drill bit–rock interaction model by studying the hole-bottom rock mechanics of air-hammer drilling, established a damage evolution equation for rock unit strength degradation, coupled it relationally with the HJC constitutive mode, and proposed a method to evaluate the rock-breaking performance of simulated downhole drill bits. Gong et al.^8^. proposed a method to obtain the HJC constitutive model of coal rocks through mechanical experiments combined with LS-DYNA finite element software. Yuan et al.^9^. studied the stress uniformity of rock specimens by adjusting the non-parallelism of the ends of the rock specimens, and performed numerical simulations based on the HJC constitutive model, which showed that the non-parallelism of the end of rock specimens should be limited to 0.10%. Ling et al.^10^. determined the parameters of a sandstone HJC constitutive model based on indoor test results and compared the simulation results with the test results; the rationality of the parameter determination method was verified. Zhang et al.^11^ combined the HJC constitutive model with smooth particle hydrodynamics (SPH) to simulate the three-dimensional invasion of granite slabs at an initial rate of 0–4000 m/s. Liu et al.^12^. proposed an improved HJC constitutive model of the tensile strength of rock materials, which can describe the tensile response of rock materials under static and dynamic loading more effectively than the traditional constitutive model, and verified the applicability of the improved HJC constitutive model via field testing and numerical simulation. Xie et al.^13^. proposed four different numerical models of composite coal rocks and, based on the HJC constitutive model, studied the stress waveform via impact testing of composite coal rock. They found that the bonding of coal rock is mainly broken by the coal body, and is independent of the impact velocity and bonding method. Wang et al.^14^. created a constitutive model of rock materials that was better able to describe the tensile damage to rock materials by coupling additional damage criteria, and the results showed that the improved constitutive model could accurately simulate the formation of fracture zones and the propagation of tensile cracks in a single blast problem. Li et al.^15^. determined the parameters of an HJC constitutive model of marble after the high temperature was determined. The variation of the stress–strain curve of marble after the high temperature was revealed. Tian et al.^16^. solved the problems of unclear characteristics of compaction stages and peak stress prediction in limestone under impact loading by revising the equation of state of the HJC constitutive model, and proposed a method for determining the parameters of the modified HJC constitutive model for limestone. Hu et al.^17^. improved the HJC constitutive structure model using the unified strength theory as the strength criterion. They introduced two damage variables, i.e., compression and tension, to characterize the damage differences between compression and tension, and verified the reliability and accuracy of the improved HJC constitutive model using four different loading conditions.

There is no doubt that scholars have contributed to the improvement and determination of parameters of the HJC constitutive model for homogeneous rock masses, but there is still a lack of research regarding jointed rock masses. Therefore, some scholars have carried out research on jointed rock masses. Zhang et al.^18^. linked the calculation of the blasting block with the blasting mechanism of a jointed rock mass, established a rock-mass damage mechanics model, and proposed the "rock fracture ratio". Ju et al.^19^. conducted SHPB tests on rock specimens with different roughness joints in respect of the energy dissipation and fractal dimension. Tsubota et al.^20^. performed dynamic experiments with naturally jointed Ryoke gneisses and revealed the effects of rock roughness, hardness, and weathering on the shear resistance of rocks; however, their conclusions apply only to Ryoke gneisses. Liu et al.^21^. used precast joint specimens to reveal the relationship between jointed rock masses and dynamic strength and damage modes. Niktabar et al.^23^. tested the shear strength of joints with different rough undulation angles and concluded that these angles are positively correlated with the shear strength of the joints. Walton et al.^24^. analyzed the relationship between key parameters such as peak strength, residual strength, and stiffness of the rock, and pre-set parameters in the form of seams. Wang et al.^25^. used the HJC constitutive model to study the damage evolution pattern of intact and jointed rock masses, and the results showed that the damage distribution exhibits a reverse S-shaped attenuation with the increase in the distance from the borehole. Huang^26^ conducted a study on jointed rock masses based on the fluid–solid coupling method and the HJC constitutive model, and suggested that the presence of joints would significantly affect the propagation of blast waves in the rock masses. Pan et al.^27^. studied the influence of joint angles on the dynamic response characteristics of rock materials, such as dynamic strength, energy dissipation, and microscopic damage. Chen et al.^28^. performed a numerical simulation of joints under different rock static pressures, carried out using a discrete element program, and the influence of joint inclination and static rock pressure on the stress–strain curves, rupture patterns, and contact force distribution of joint specimens was revealed. Wang et al.^29^. tested the cyclic loading and unloading of jointed sandstone with different limit stress ratios, and proposed that when the limit stress ratio of jointed sandstone is greater than or equal to 0.90, jointed sandstone is damaged during circulation and fatigue frequency decreases with the increase in the limit stress ratio. Liu et al.^30^. determined the HJC constitutive model parameters of layered slate through indoor tests and proposed a hole placement method for large section tunnel blasting. Tian et al.^31^. determined the HJC constitutive model parameters of 60° layered slate through indoor tests and carried out a study on the hole placement method of tunnel blasting under the effect of geostress. The above two scholars spent a lot of time and expense in the early stage of the research to conduct indoor tests on laminated slate and determine the parameters of the HJC constitutive model. It is necessary to propose a high-precision numerical simulation method as soon as possible to replace the indoor test, to ensure the accuracy of numerical simulation while improving the efficiency and saving the cost.

Current research focuses on the analysis of the HJC constitutive model of slate with special joint angles, but the joint angles of natural slate are often not special, and the method of determining their HJC constitutive model parameters is rarely reported. Therefore, in this study, we took the Tongzi tunnel in the expanded section of the Chongqing-Zunyi Expressway in China as the research background, drilled core samples on site, and conducted indoor tests to obtain the static dynamic parameters of the jointed slate. Based on the results of the indoor tests, the parameters of the HJC constitutive model of the slate at a special joint angle were determined by combining the model with the sensitivity analysis method. Based on the HJC constitutive model parameters of the slate at the special joint angle, the variation law of the HJC constitutive model parameters with the joint angle of the slate was analyzed. The fitting relationship between the HJC constitutive model parameters and the joint angle of the slate was determined, and a numerical simulation method for the dynamic impact of the slate at the natural joint angle was proposed.

## Determination of the HJC constitutive model parameters for joint angles

The HJC constitutive model is a concrete inherent model proposed by Holmquist, Johnson, and Cook. The HJC model consists of the state equation, yield surface equation, and damage evolution Eq. ^32^. The model contains 21 parameters divided into density *ρ*, uniaxial compressive strength *f*_*c*_, tensile strength *T*, shear modulus *G*, characteristic cohesive strength *A*, pressure hardening factor *B*, standardized pressure hardening index *N*, strain rate influence factor *C*, characteristic maximum strength *SF*_*max*_, damage parameters *D*_*1*_ and *D*_*2*_, minimum plastic strain *EF*_*min*_ at material failure, pressure constants *K*_*1*_, *K*_*2*_, *K*_*3*_, crushing pressure *P*_*c*_, crushing volume strain *μ*_*c*_, ultimate hydrostatic pressure *P*_*l*_, ultimate hydrostatic volume strain *μ*_*l*_, reference strain rate *EPSO*, and failure type *F*_*S*_^16^. Hydrostatic tests and SHPB tests test were carried out to calculate the parameters of the slate HJC model.

### Static test

**Figure 1:**
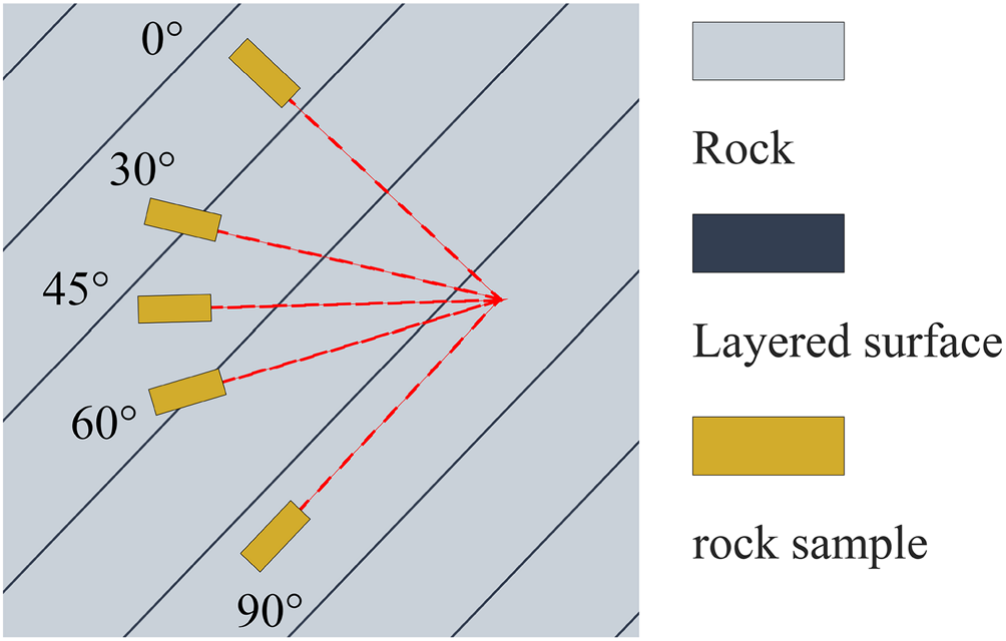
Schematic diagram of on-site coring.

**Figure 2:**
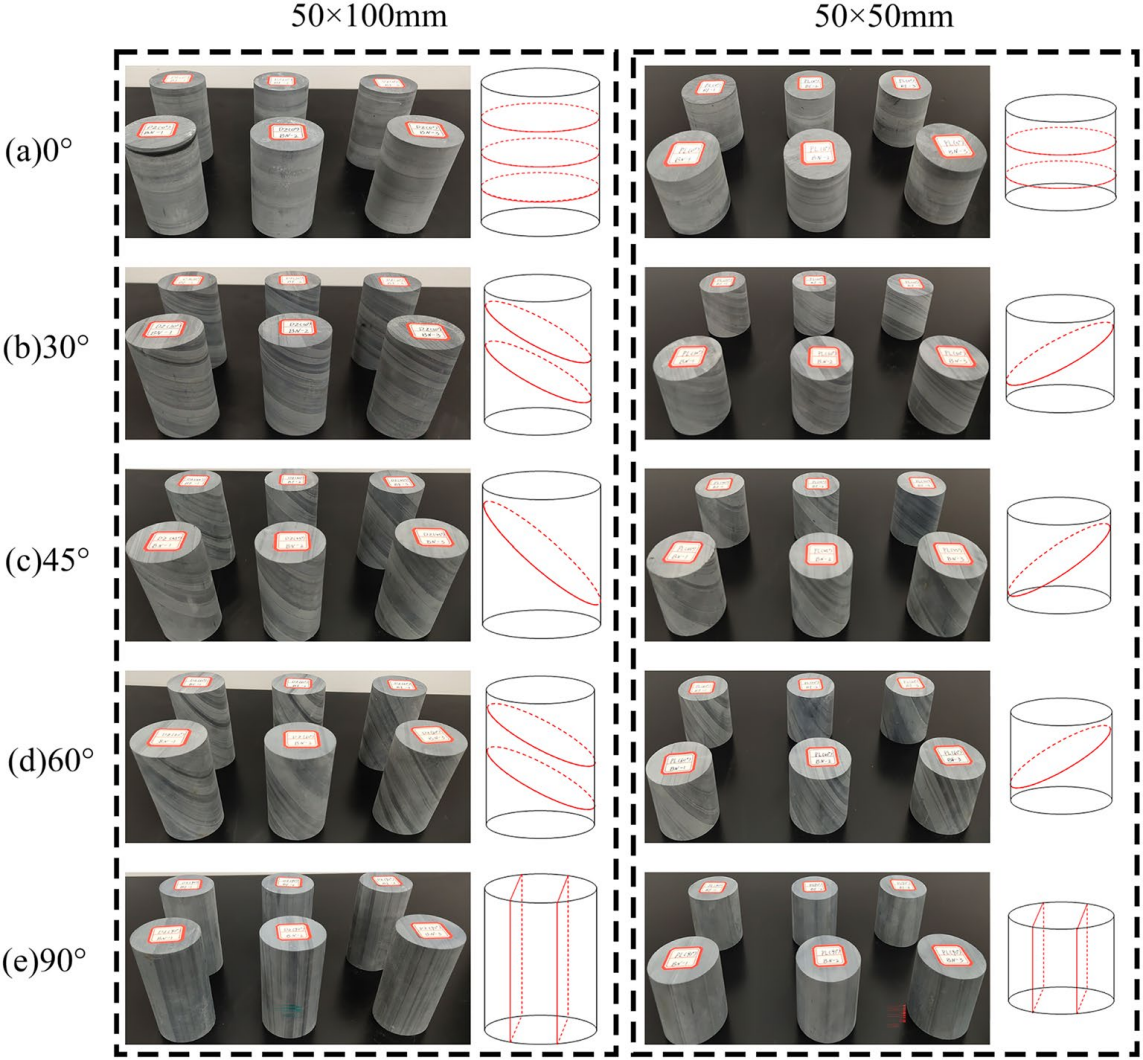
Standard rock sample of slate.

In this study, the uniaxial compression test, triaxial compression test, and Brazilian splitting test were carried out, using the Tongzi tunnel in Guizhou, China, as the engineering background, and on-site core drilling and sampling. Considering that in the Trigonometric functions, 0°, 30°, 45°, 60° and 90° have special values, this paper uses these five angles as different inclination variables, based on these angles, nine samples for each angle were obtained. In order to avoid errors in test results due to sample dispersion, all rock samples were taken from the same rock mass. Samples were obtained according to the International Society for Rock Mechanics (ISRM) requirements for rock sample sizes in static samples^33^. Field drill core sampling is shown in Fig. 1. The rock was processed and polished into φ50 × 100mm and φ50 × 50mm cylindrical specimens. The flatness of both ends of the specimen did not exceed 0.02mm. The sides were smooth and straight to meet the requirements of verticality, and the slate samples are shown in Fig. 2.

**Figure 3:**
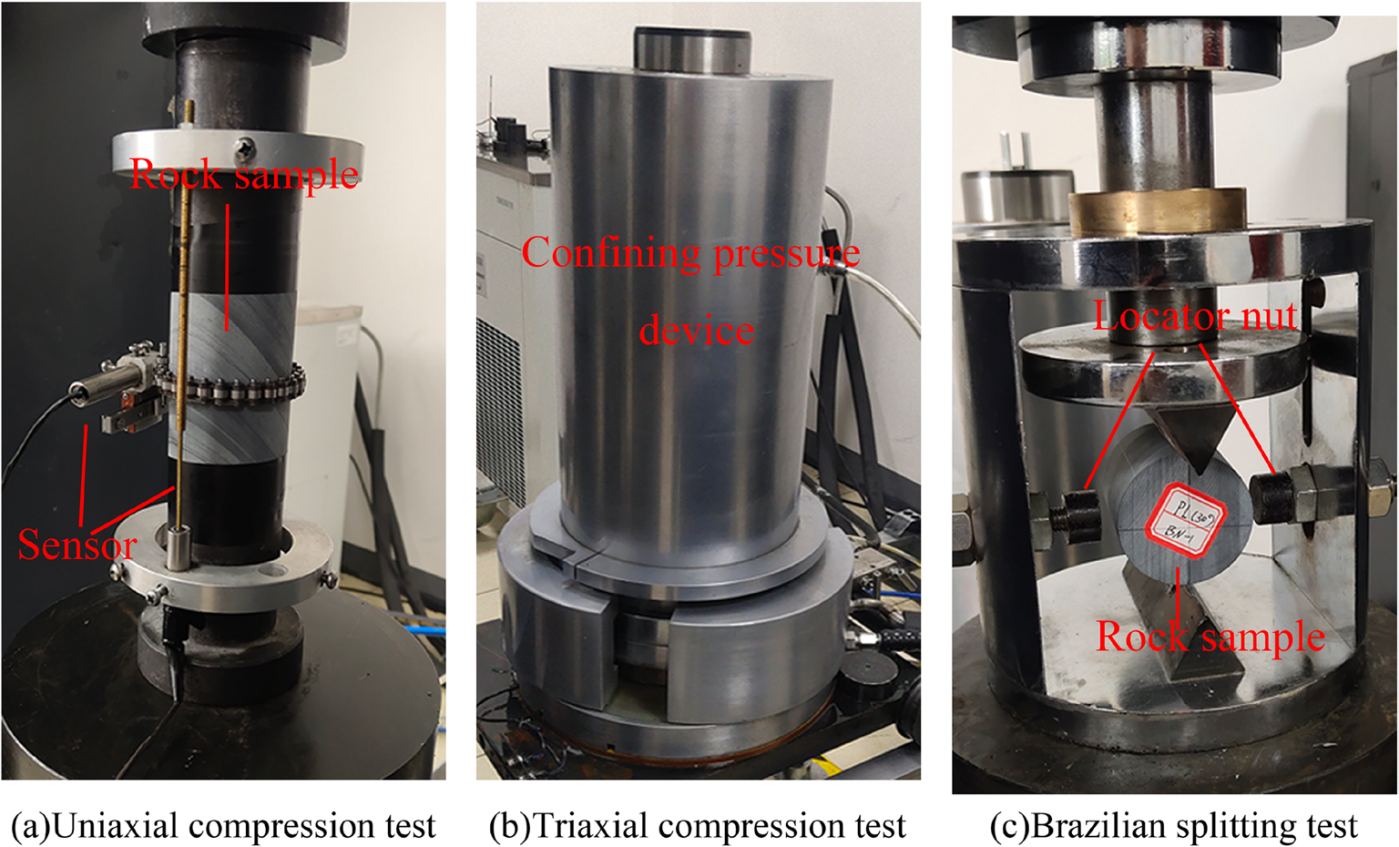
Static test. (**a**) Uniaxial compression test; (**b**) triaxial compression test; (**c**) Brazilian splitting test.

The diameter and height of rock samples were measured using a Vernier caliper, and the average density of rock samples was calculated at 2754 kg/m^3^ after weighing rock samples on an electronic scale. A microcomputer-controlled electro-hydraulic servo rock three-axis testing machine controlled by a TAJW-2000 microcomputer was used to carry out the static test. The uniaxial compression test used a displacement rate 0.005 mm/s, during which transverse and axial sensors were installed in the middle of the sample to measure the transverse and axial strain compression processes. The triaxial compression test applied surrounding pressures of 5 MPa, 10 MPa, and 15 MPa to the rock samples using the same loading method as the single-axis test. When conducting the Brazilian splitting test, we first drew two positioning aid lines through the center of the end-face of the rock sample, then placed the rock sample in the fixture, rotating the positioner nut so that the upper and lower cutter mouths were located on the positioning aid line. We loosened the positioner nut, then the concentrated load on the bearing platform was transformed into a line load on the rock sample through the fixture, resulting in the destruction of the rock sample due to tension. The test procedure is shown in Fig. 3.

Shear modulus *G* and volume modulus *K* were calculated using *G* = *E*/2 (1 + *μ*) and *K* = *E*/3 (1-2*μ*), respectively. The static parameters of the slate are shown in Table 1.

**Table 1:** Static parameters of slate.

Angle/°	Density ρ/(kg/m^3^)	Uniaxial compressive strength *f*_*c*_/MPa	Triaxal compression strength		Modulus of elasticity *E*/GPa
			*σ*_*2*_ = *σ*_*3*_ (MPa)	*σ*_*1*_ (MPa)	
0	2754	142.16	5	179.16	25.33
			10	197.60	
			15	208.99	
30		58.81	5	62.06	18.11
			10	70.67	
			15	81.04	
45		38.82	5	45.74	20.80
			10	52.87	
			15	58.63	
60		31.43	5	31.43	24.83
			10	35.64	
			15	40.47	
90		136.14	5	127.24	41.8
			10	144.62	
			15	165.98	

Angle/°	Poisson ratio *μ*	Shear modulus* G*/GPa	Bulk modulus *K*/GPa	Tensile strength *T*/MPa	
0	0.16	10.92	12.42	18.31	
30	0.17	7.74	9.15	13.08	
45	0.16	8.97	10.20	10.98	
60	0.24	10.01	15.92	8.08	
90	0.27	16.46	30.29	1.22	

### SHPB test

**Figure 4:**
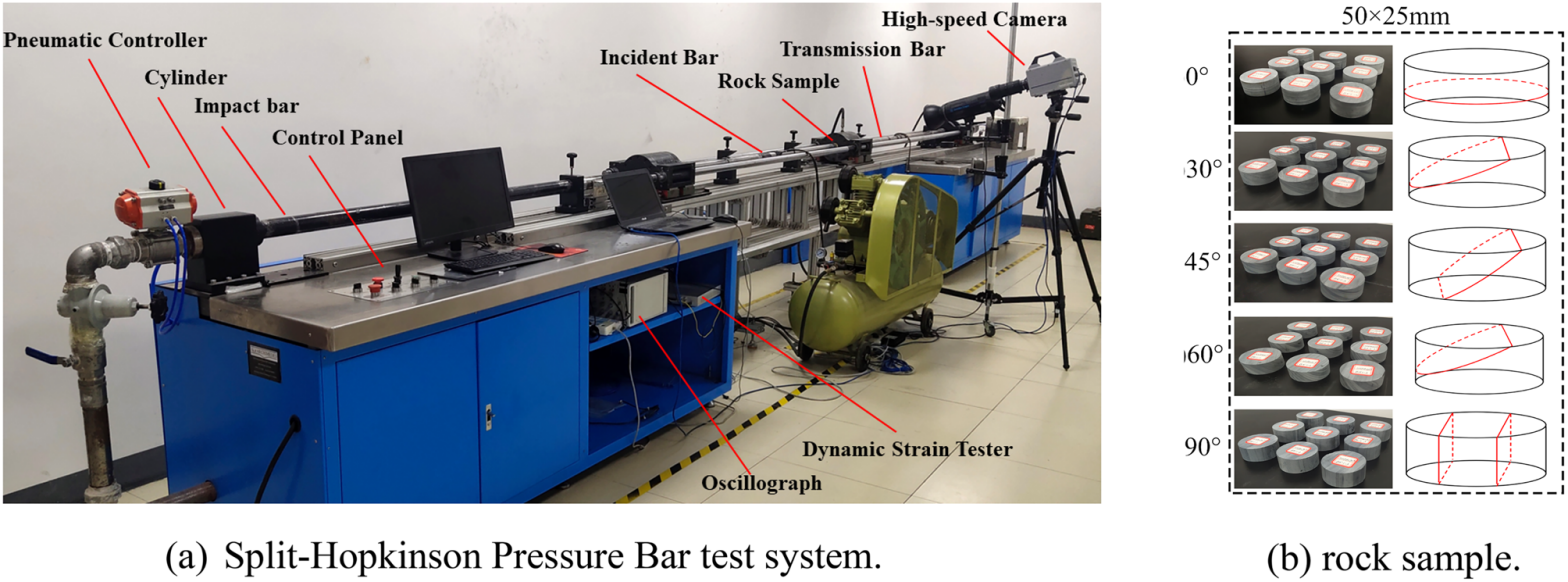
Test system and rock specimen. (**a**) Split-Hopkinson pressure bar test system; (**b**) rock sample.

The SHPB test used a standard cylindrical specimen of φ50 × 25mm, and the test device was the Hopkinson compression rod ALT100 test system, which comprises an active console module, a compression rod module, and a data acquisition module. The compression rod module consists of bullets, incidence rods, transmission rods, and energy-absorbing rods; the relevant parameters of the compression rod module are shown in Table 2. The data acquisition system consists of strain gauges, a Wyeth bridge circuit, a hyperdynamic strain gauge, and a hypervelocity camera. The test equipment is shown in Fig. 4.

**Table 2:** Basic parameters of SHPB test system.

Parameters	Parameter value	Parameters	Parameter value
Diameter	50 mm	Density	7.81 g/cm^3^
Bullet length	400 mm	Modulus of elasticity	210 GPa
Incident rod length	2000 mm	Poisson ratio	0.28
Transmissive rod length	2000 mm	Longitudinal wave speed	5410 m/s

Dynamic impact tests were performed on the slate at different nodal dips using impact pressures of 0.1 MPa (impact velocity of approximately 6.86 m/s), 0.2 Mpa (impact velocity of approximately 13.74 m/s), and 0.3 Mpa (impact velocity of approximately 20.61 m/s), with the results of an average of three tests per set shown in Table 3.

**Table 3:** Experimental results of dynamic compression of slate at different angles.

Number	Impact strength/(MPa)	Nodal angle/(°)	Peak stress/(MPa)
1	0.1	0	133.77
2	0.2		139.10
3	0.3		156.54
4	0.1	30	92.13
5	0.2		111.92
6	0.3		132.76
7	0.1	45	70.42
8	0.2		86.09
9	0.3		98.09
10	0.1	60	74.22
11	0.2		97.19
12	0.3		114.69
13	0.1	90	154.54
14	0.2		161.48
15	0.3		169.97

### Parameter determination

The model strength of HJC material^32^ is expressed in Eq. (1), which ignores the effects of damage, strain rate, and temperature, and can be reduced to Eq. (2) in a natural state.

1 $$ \sigma^{*} = \left[ {{\text{A}}(1 - {\text{D}}) + {\text{BP}}^{{* {\text{N}}}} } \right]\left[ {1 + {\text{C}}\ln \left( {\varepsilon^{*} } \right)} \right] $$

2 $$ \sigma^{*} = {\text{A}} + {\text{BP}}^{{* {\text{N}}}} $$

where σ^*^ is the dimensionless equivalent force; P^*^ is the dimensionless static pressure, calculated as *A* = *c*/*f*_*c*_ (1 + *C*Ln10^–4^).

**Figure 5:**
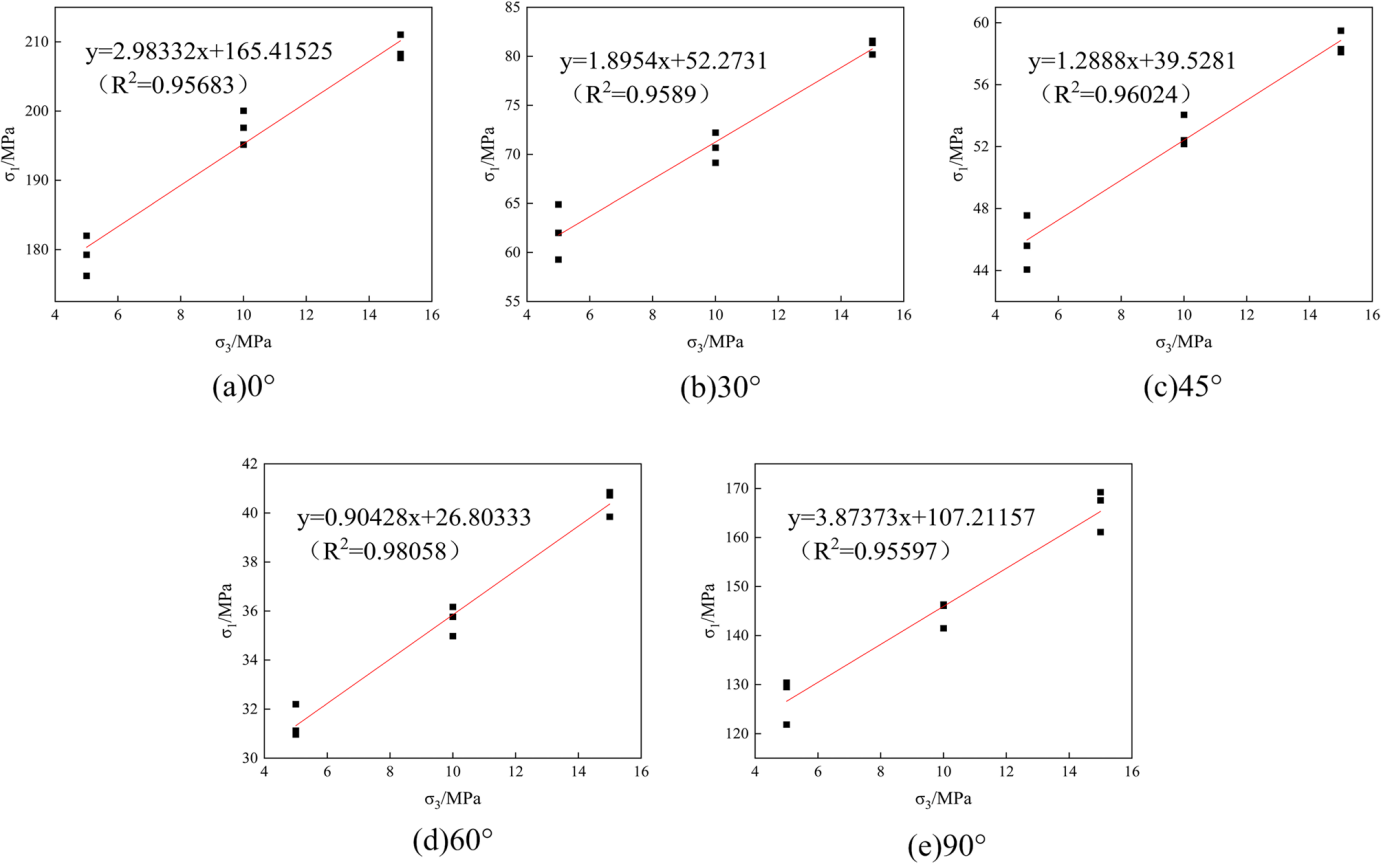
Axial pressure–perimeter pressure relationship. (**a**) 0°; (**b**) 30°; (**c**) 45°; (**d**) 60°; (**e**) 90°.

**Figure 6:**
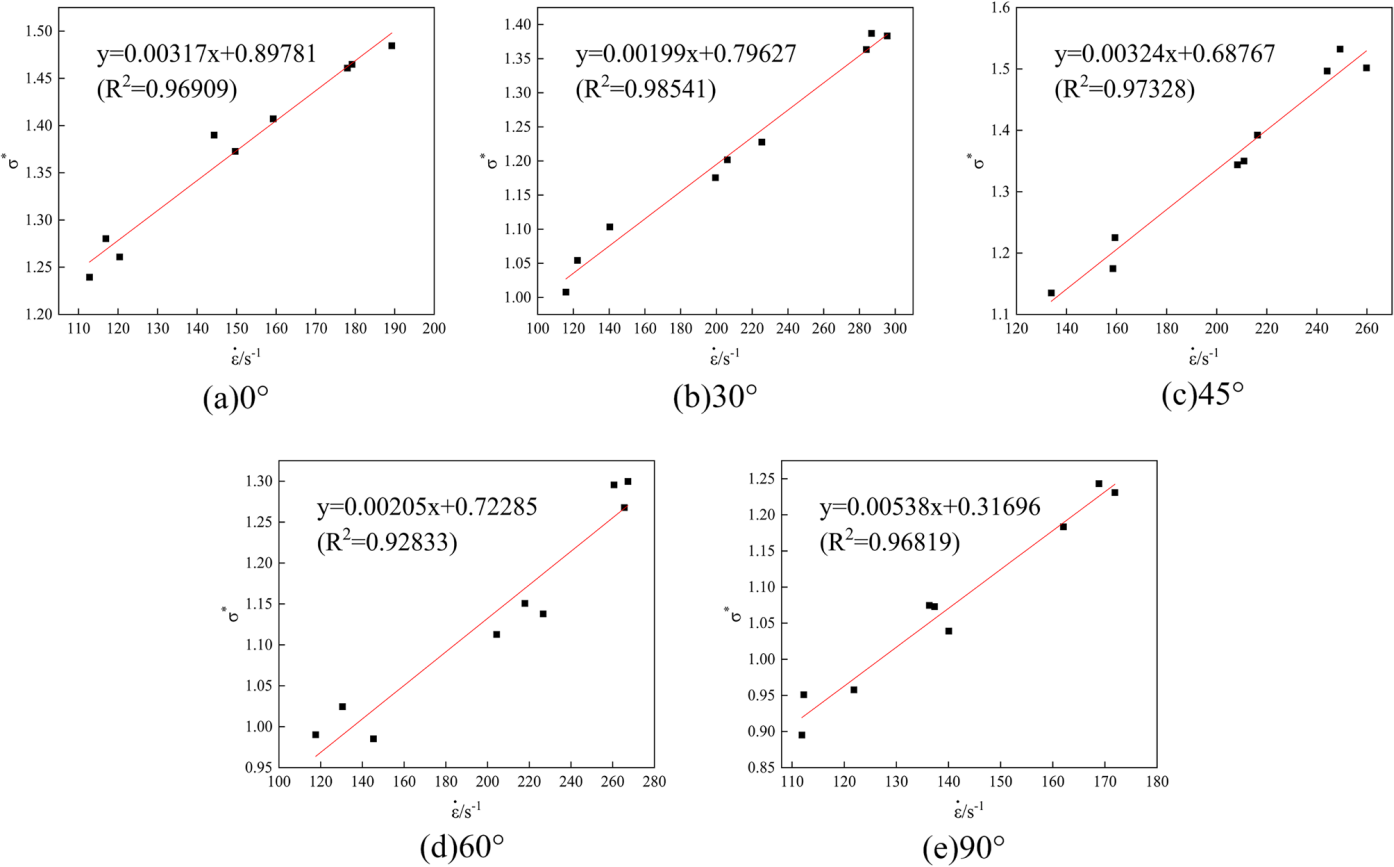
Parameter *C* fit. (**a**) 0°; (**b**) 30°; (**c**) 45°; (**d**) 60°; (**e**) 90°.

**Figure 7:**
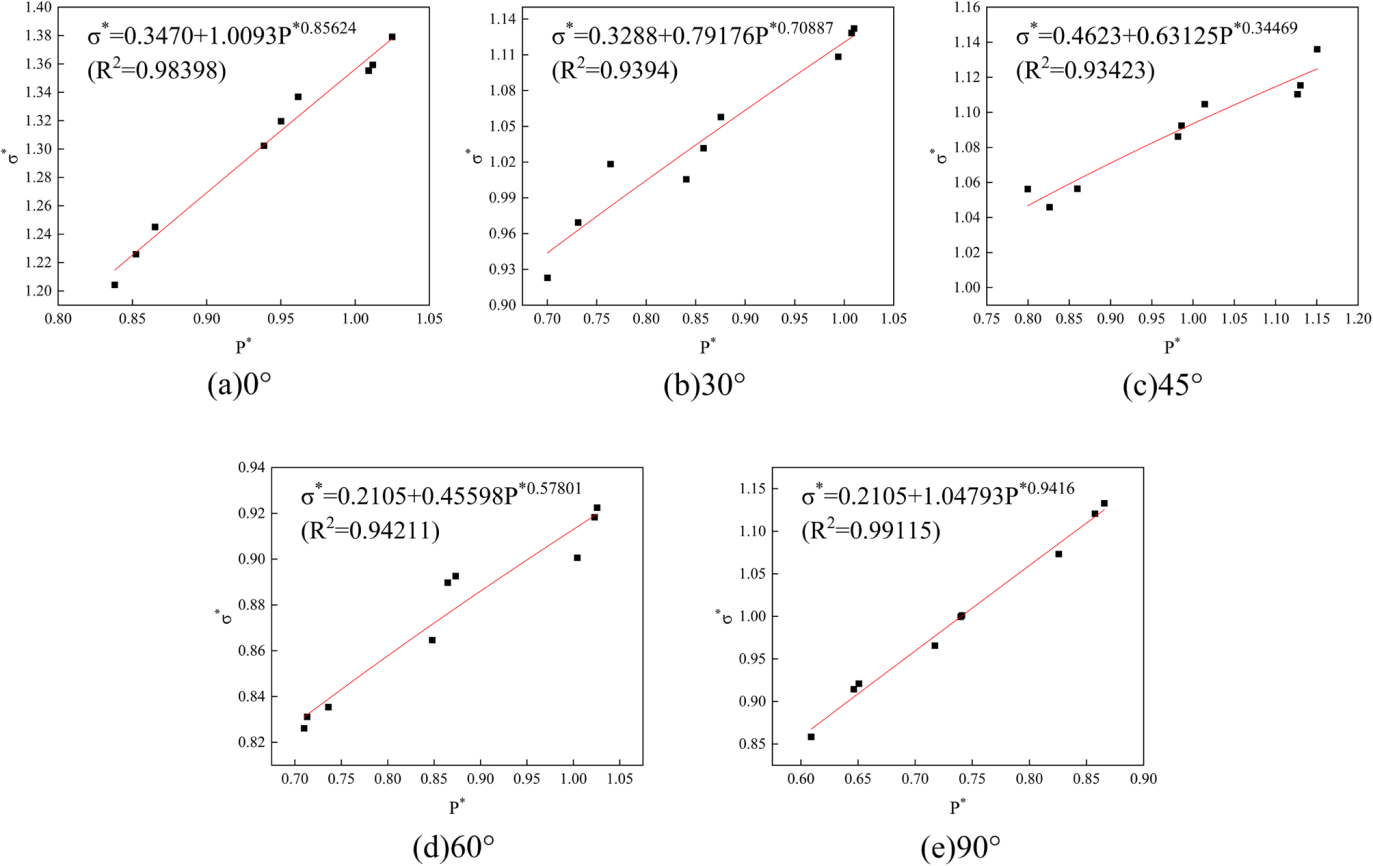
Parameter *B*, with *N* values of (**a**) 0°; (**b**) 30°; (**c**) 45°; (**d**) 60°; (**e**) 90°.

The cohesion *c* of the rock sample was calculated based on the results of triaxial compression tests with ambient pressures of 5 MPa, 10 MPa, and 15 MPa, combined with M-C criterion^34^, *c* = *σ*_*c*_(1-sin*φ*)/2cos*φ*, in which *φ* is the angle of friction, *φ* = arcsin(*m*-1/*m* + 1), and *m* is the slope of the axial ring pressure relationship graph, as shown in Fig. 5. Based on the results of the SHPB dynamic impact test, the strain rate and the characterized strength were fitted in combination with the calculation method adopted by Tian et al.^35^, as shown in Fig. 6, to derive the value of the strain rate influence factor *C*. The values of the characteristic pressure hardening factor *B* and the normalized hardening index *N* were obtained by substituting the characteristic cohesive strength *A* already obtained into the curve fitted using Eq. (2), as shown in Fig. 7. According to Fang et al.^5^, *SF*_*max*_ and the reference strain rate *EPSO* were determined to be 20.0 and 1.0, respectively.

The damage parameter *D*_*1*_ was determined using the equation *D*_*1*_ = 0.01/(1/6 + T^*^), with *D*_*2*_ constant at 1.0, and the fault type *F*_*S*_ reference value^5^ at -0.001.

The crushing pressure *P*_*c*_, crushing volume strain *μ*_*c*_, and ultimate hydrostatic volume strain *μ*_*l*_ were calculated using *P*_*c*_ = *f*_*c*_/3, *μc* = *P*_*c*_/*K*, and *μ*_*l*_ = *ρ*_*g*_/*ρ*_*0*_-1, respectively, where *p*_*g*_ is the compaction density, which was taken as 2900 kg/m^3^.

The pressure constants *K*_*1*_, *K*_*2*_, *K*_*3*_, and the compaction limit hydrostatic pressure *P*_*l*_ were extracted from the results of a study in the literature^5^.

The parameters of the HJC constitutive model of slate with a special joint angle are shown in Table 4.

**Table 4:** Slate HJC model parameter values.

Angle	*ρ*/(kg·m^−3^)	*G*/GPa	*A*	*B*	*C*	*N*	*f*_*c*_/MPa	*T*/MPa	EPSO	*Ε*_*f,min*_
0°	2754	10.92	0.3470	1.0093	0.0032	0.8562	142.16	18.31	1	0.01
30°		7.74	0.3288	0.7918	0.0020	0.7089	58.81	13.08		
45°		8.97	0.4623	0.6313	0.0032	0.3447	38.82	10.98		
60°		10.01	0.4570	0.4560	0.0021	0.5780	31.43	8.08		
90°		16.46	0.2105	1.0479	0.0054	0.9416	136.14	1.22		

*S*_*max*_	*P*_*C*_/GPa	*μ*_*c*_	*P*_*l*_/GPa	*μ*_*l*_	*D*_*1*_	*D*_*2*_	*K*_*1*_/GPa	*K*_*2*_/GPa	*K*_*3*_/GPa	*F*_*s*_
20	0.0474	0.0038	2.02	0.053	0.034	1	39	− 223	550	− 0.001
	0.0196	0.0021			0.026					
	0.0129	0.0013			0.022					
	0.0105	0.0007			0.024					
	0.0454	0.0015			0.057					

### Sensitivity analysis

Sensitivity analysis of model parameters is a method used to study the variation of parameters in the calculation results. Sensitivity analysis can effectively identify key parameters that have a significant impact on the model calculation. There are multiple parameters of the HJC constitutive model of rocks, and the values of these parameters for different types of rocks are different. In order to improve the accuracy of numerical simulation, it is necessary to analyze each parameter. In this study, the parameters of the constitutive model of slate HJC were analyzed using sensitivity analysis^36^.

For the SHPB numerical model, the maximum peak *σ*_*max*_ stress of the rock sample is the target function, and slate is the object of study. Let the objective function peak stress *σ*_*maxi*_ be mainly influenced by n parameters *F*_*1*_, *F*_*2*_, *F*_*3*_, ….. , *F*_*n*_. *σ*_*max*_ = *f*(*F*_*1*_, *F*_*2*_, *F*_*3*_, ….. , *F*_*n*_). The benchmark parameter *F*_i_^*^ is set, and the evaluation index is σ^*^_max_. For the parameters in the HJC constitutive model, the parameters in Table 4 are set as the benchmark parameters, and four values of − 40%, − 20%, + 20%, and + 40% are selected on the benchmark parameters for the numerical simulation and calculation analysis.

The rate of change of parameter *δF*_*ij*_ is expressed as

3 $$ \delta F_{ij} = \frac{{F_{ij} - F_{i}^{*} }}{{F_{i}^{*} }} \times 100\% (i = 1,2,3 \ldots \ldots n;j = 1,2,3 \ldots \ldots n) $$

where *F*_*ij*_ is the j-th parameter derived from the i-th parameter in the HJC constitutive model based on the adjustment of the reference parameter *F*_i_^*^.

*δF*_*ij*_ (*j* = 1, 2, 3, 4) corresponds to − 40%, − 20%, 20%, 40%.

Peak stress variations in *δσ*_*maxij*_ are expressed as

$$ \delta \sigma_{{\text{maxij}}} = \frac{{\sigma_{{\text{maxij}}} - \sigma_{{\max^{*} }} }}{{\sigma_{{\max^{*} }} }} \times 100\% (i = 1,2,3 \ldots \ldots n;j = 1,2,3,4) $$

where *σ*_*maxij*_ denotes the peak stress obtained by simulating the i-th parameters of the HJC constitutive model at the j-th parameter change rate.

**Figure 8:**
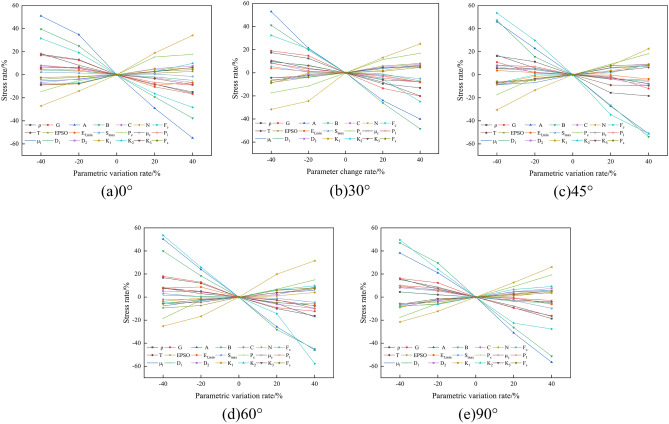
Sensitivity analysis of peak stress to HJC parameters. (**a**) 0°; (**b**) 30°; (**c**) 45°; (**d**) 60°; (**e**) 90°.

Based on the results of a large number of numerical simulations, the peak stress change rate caused by the variation of parameters in the HJC constitutive model of slate was obtained, as shown in Fig. 8.

As can be seen from Fig. 8, the parameters *ρ*, *G*, *A*, *B*, *N*, *F*_*c*_, *P*_*c*_, and *μ*_*c*_ have a large effect on the peak stress, and the absolute value of the rate of change of the peak stress in the parameter interval is more than 10%; the changes in parameters *A* and *B* make the change in the peak stress close to 60%, and the absolute values of the remaining parameters on the rate of change of the peak stress are all less than 10%. The literature^36^ shows that a parameter is sensitive when the absolute value of the change rate of the target function is greater than 10%. Therefore, parameters such as *ρ*, *G*, *A*, *B*, *N*, *F*_*c*_, *P*_*c*_, and *μ*_*c*_ can be considered sensitive to peak stress during slate failure.

## Parameter verification

### Modeling

**Figure 9:**
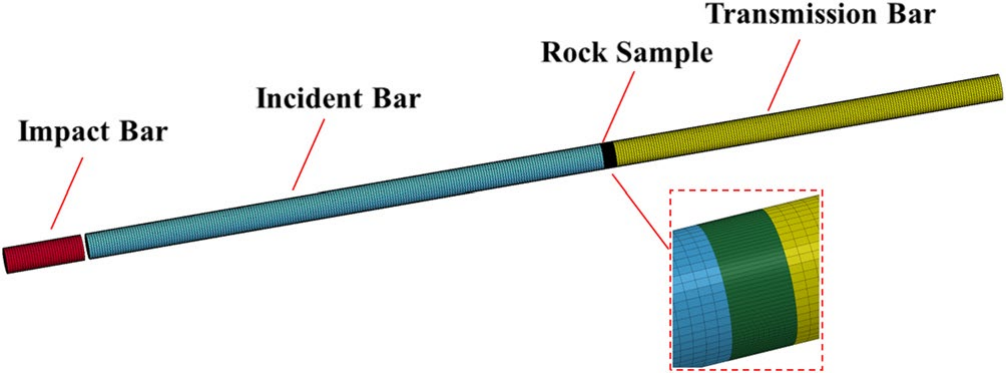
Numerical simulation model diagram.

We used Hypermesh 2019 software to create a numerical model of the SHPB dynamic impact, as shown in Fig. 9. In order to ensure the accuracy of the rock calculation, the grid size of the rock specimen was set at 1mm and divided into 50 parts along the radial direction. Since the impact bar, incident bar, and transmission bar do not require high accuracy, the grid size was set at 5 mm and divided into 200 parts along the radial direction. We selected MAT_ ELASTIC for the impact stick, incident bar stick, and transmission bar materials in ANSYS/LS-DYNA software. The impact bar, incident bar, and transmission bar were assigned values using the parameters in Table 2, and samples were selected in ANSYS/LS-DYNA with a material of MAT_ JOHNSON_ HOLMQUIST_ CONCRETE^37^.

The HJC constitutive model parameters in Table 4 were used for numerical simulation calculations, and the material properties of the impact bar, incident bar, rock specimen, and transmission bar were defined by modifying the K file. Contact setting was performed using CONTACT_ AUTOMATIC_ SURFACE_ TO_ SURFACE in ANSYS/LS-DYNA. INITIAL_ VELOCITY_ GENERATION was used to assign the impact velocity to the bullet and to add the tensile failure criterion MAT _ADD_ EROSION for rocks in the K file^37^.

### Verification of force state

Based on the stress balance assumption and the one-dimensional stress-wave theory, the incident, reflection, and transmission signals of the rock were measured, and the dynamic stress, strain, and strain rate of the rock specimens were calculated accurately via the three-wave method^38^. The calculation equations are as follows.

5 $$ \sigma (t) = \frac{{A_{0} E}}{2A}\left[ {\varepsilon_{I} (t) + \varepsilon_{R} (t) + \varepsilon_{T} (t)} \right] $$

6 $$ \varepsilon (t) = - \frac{{\text{c}}}{{\text{L}}}\int_{0}^{{\text{T}}} {\left[ {\varepsilon_{{\text{I}}} ({\text{t}}) + \varepsilon_{{\text{R}}} ({\text{t}}) - \varepsilon_{{\text{T}}} ({\text{t}})} \right]} $$

7 $$ \dot{\varepsilon }({\text{t}}) = - \frac{{\text{c}}}{{\text{L}}}\left[ {\varepsilon_{{\text{I}}} ({\text{t}}) + \varepsilon_{{\text{R}}} ({\text{t}}) - \varepsilon_{{\text{T}}} ({\text{t}})} \right] $$

where $\varepsilon_{I} (t)$ is the incident signal; $\varepsilon_{R} (t)$ is the reflection signal; $\varepsilon_{T} (t)$ is the transmitted signal; *A* is the cross-sectional area of the specimen; *L* is the length of the specimen; *A*_*0*_ is the cross-sectional area of the compression bar; and *c* is the longitudinal wave velocity of the incident bar and transmitted bar.

**Figure 10:**
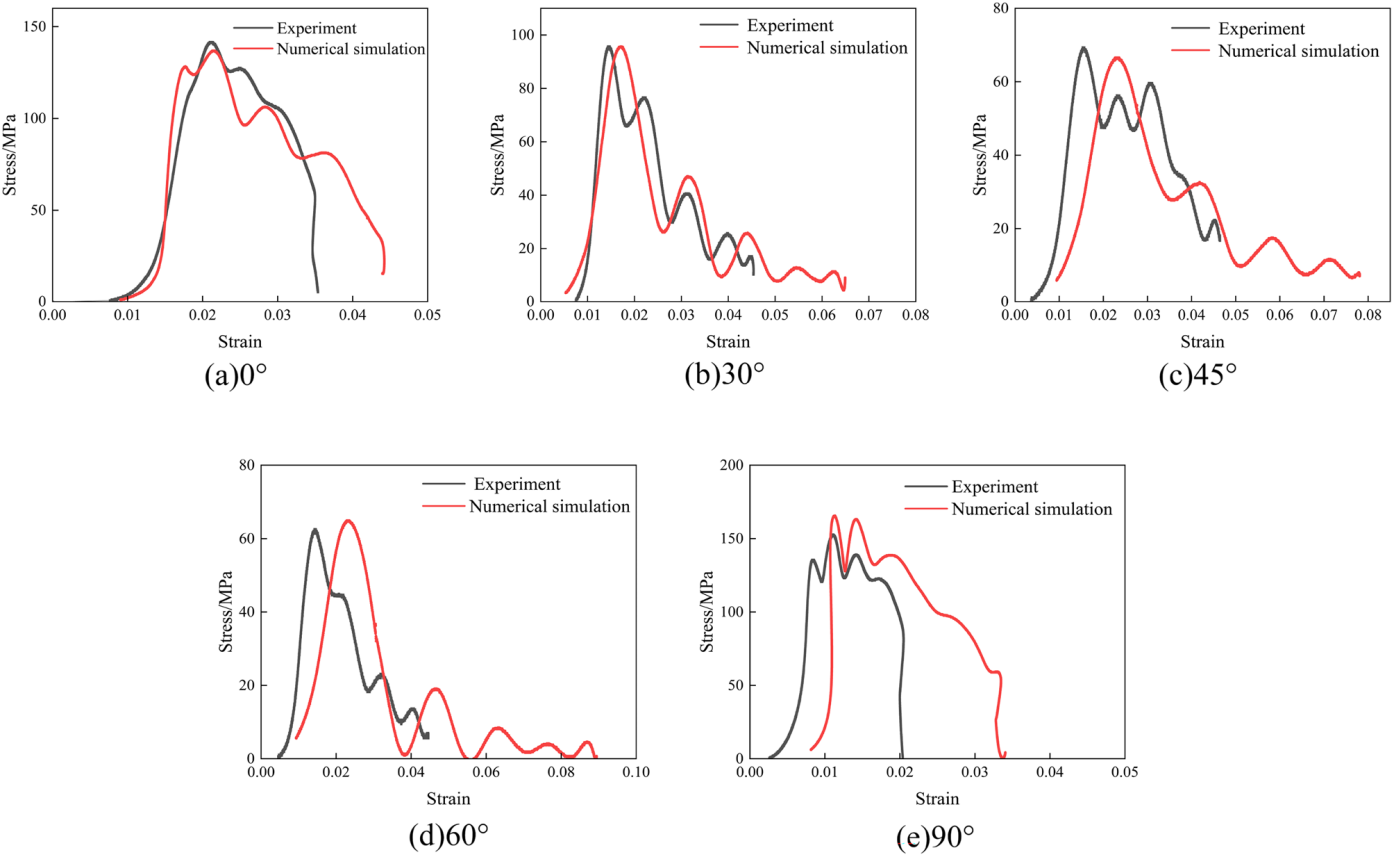
Indoor testing and numerical simulation stress–strain curves at 0.1 MPa impact pressure. (**a**) 0°; (**b**) 30°; (**c**) 45°; (**d**) 60°; (**e**) 90°.

**Figure 11:**
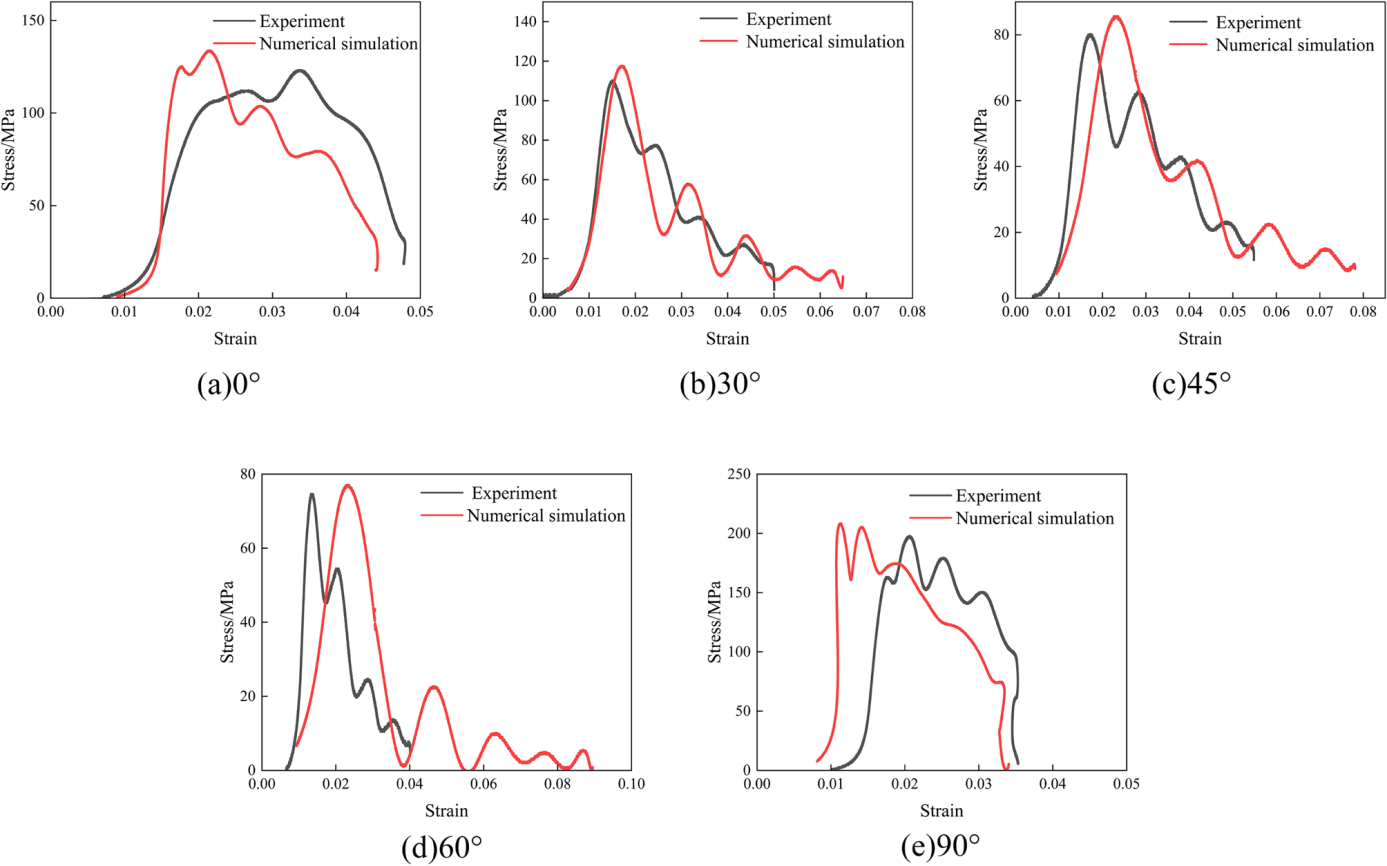
Indoor testing and numerical simulation stress–strain curves at 0.2 MPa impact pressure. (**a**) 0°; (**b**) 30°; (**c**) 45°; (**d**) 60°; (**e**) 90°.

**Figure 12:**
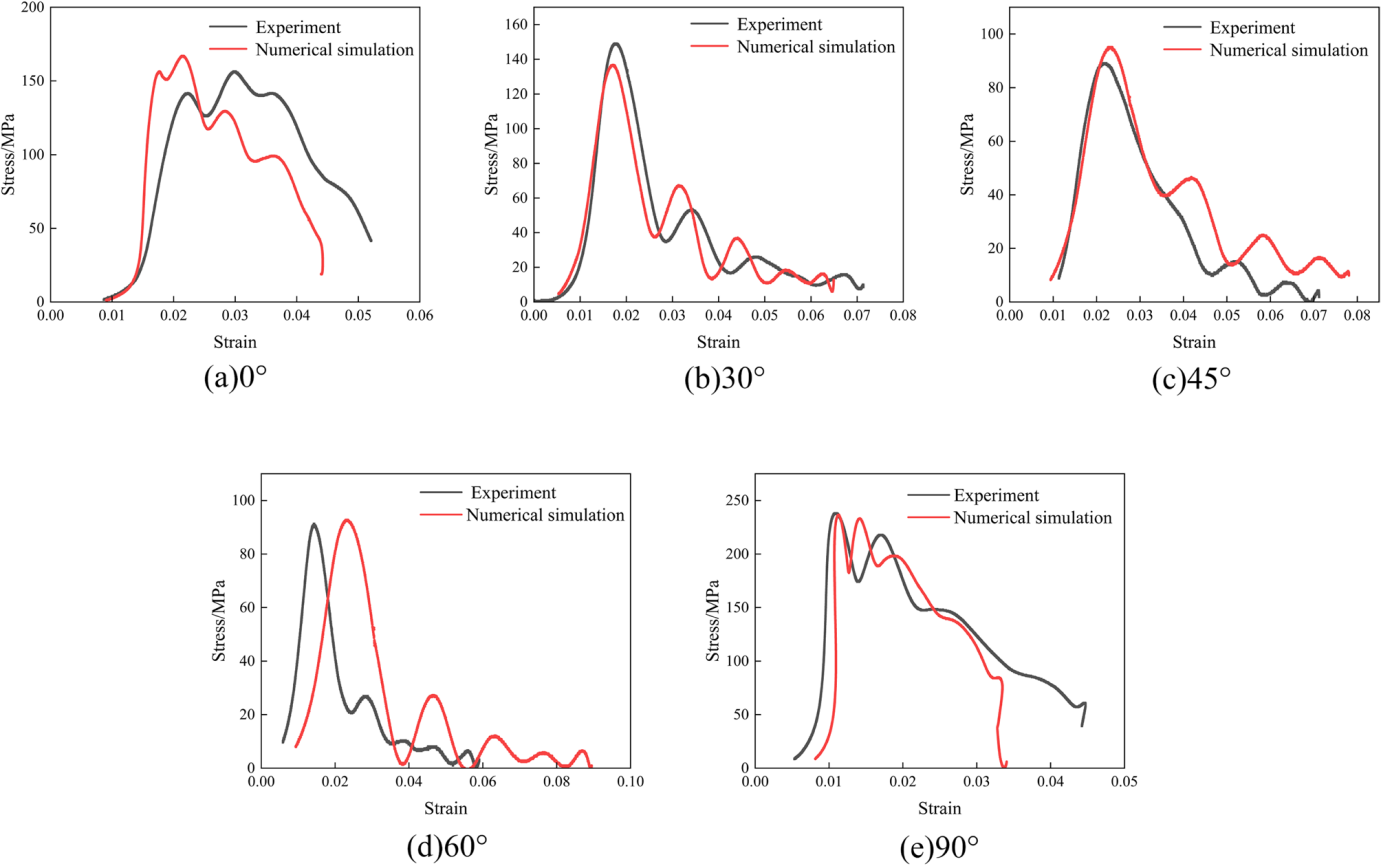
Indoor testing and numerical simulation stress–strain curves at 0.3 MPa impact pressure. (**a**) 0°; (**b**) 30°; (**c**) 45°; (**d**) 60°; (**e**) 90°.

The stress–strain curves obtained via indoor testing and numerical simulation are shown in Figs. 10, 11 and 12.

As can be seen from Figs. 10, 11 and 12, the stress–strain curves obtained via numerical simulation are similar to those obtained using indoor experiments. When the impact air pressure was 0.1MPa, the numerical simulation accuracy with joint inclinations of 0°, 30°, 45°, 60°, and 90° was 96.67%, 99.94%, 96.01%, 96.38%, and 92.25%, respectively. When the impact air pressure was 0.2MPa, the numerical simulation accuracy with joint inclinations of 0°, 30°, 45°, 60°, and 90° was 92.18%, 93.77%, 93.66%, 96.91%, and 94.93%, respectively. When the impact air pressure was 0.3MPa, the numerical simulation accuracy with joint inclinations of 0°, 30°, 45°, 60°, and 90° was 93.75%, 91.68%, 93.76%, 98.40%, and 99.37%, respectively. The average error of the above results is 4.69%. In numerical simulation, the results are related to the simplification of the mesh size and damage constitutive model theory. At the same time, the micro cracks inside the rock in indoor experiments can also cause certain errors in the test results. The combined effect of the two results in a certain error between the numerical simulation and indoor test results. Generally, an average error of within 10% is within the allowable error limit of engineering^39^. The peak stress in the rock increased and then decreased as the joint angle increased. The fluctuation in the stress–strain curve was caused by the fact that the joint damage to the rock body was earlier than the rock-body damage during the impact.

### Damage process verification

**Figure 13:**
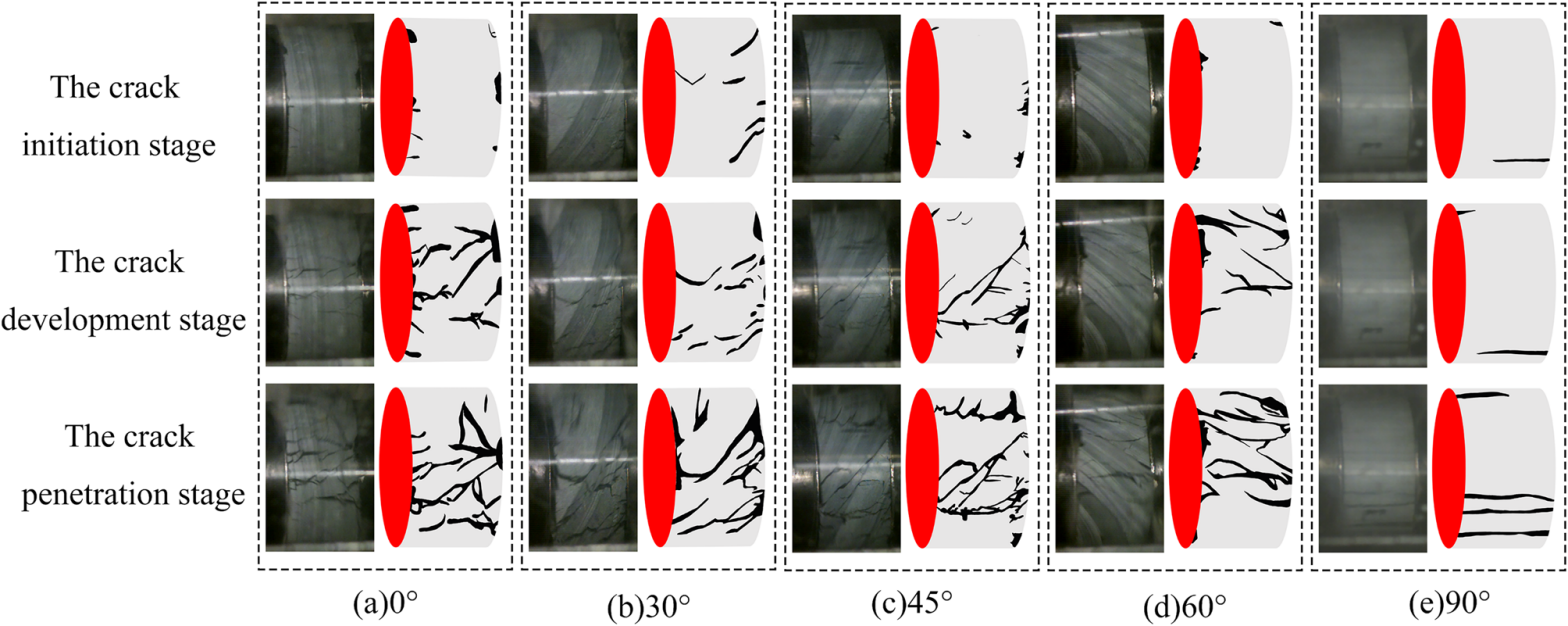
Slate destruction process for different joint angles.

The damage process for slate specimens with different nodal dips is shown in Fig. 13. It can be seen from the diagram that when the joint dip angle is 0°, the slate specimens produce fine cracks on both sides of the crack initiation stage. During the crack development stage, the fine cracks increase, the crack length increases, and during the crack penetration stage, the crack continues to increase. The fine cracks develop into penetrating cracks. When the joint angle is 30°, the slate specimen shows fine cracks at the dense joints on both sides in the stage of crack budding, and cracks appear at the joints, with vertical joints in the stage of crack development. The number of cracks continues to increase, the number of cracks increases slightly at the stage of crack penetration, and the cracks penetrate along the direction of the joints; the rock body near the cracks is crushed in the direction of vertical cracks. When the dip angle of the joints is 45°, there is no obvious crack budding and crack development in the slate specimens, most of the cracks produced directly penetrate along the direction of the joints, and the number of cracks continues to increase. When the joint angle is 60°, the damage to the slate specimens is similar to that at 30°. When the joint angle is 90°, the slate specimens show long cracks along the direction of the joints in the budding crack stage, and there is no crack development stage in the specimens with this dip angle. At the crack penetration stage, the number of cracks increases and they penetrate directly, cutting the rock samples into rock columns and causing the rock samples to be damaged by pressure.

**Figure 14:**
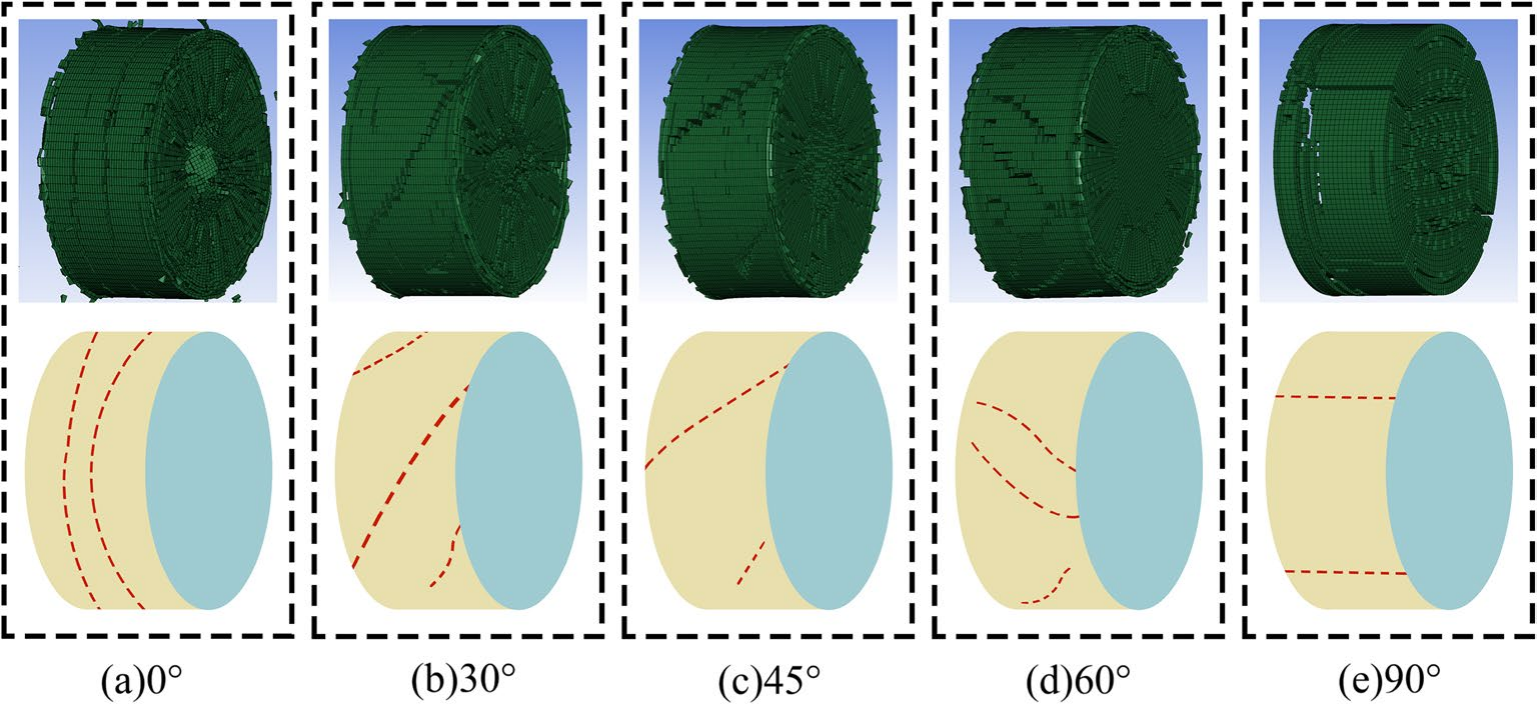
ANSYS/LS-DYNA simulation of damage process.

Material 111 MAT_ JOHNSON_ HOLMQUIST_ CONCRETE in ANSYS/LS-DYNA has its own criteria for failure. The failure criterion acts in conjunction with the rock tensile failure criterion the MAT_ ADD_ EROSION criterion, and the elements that satisfy the failure conditions are automatically deleted, leading to the formation of cracks, the increase of which leads to the failure of rock specimens. The damage process for slate specimens with different nodal dips in the numerical simulation is shown in Fig. 14.

As can be seen from Fig. 14, when the joint inclination angle is 0°, the crack direction is vertical, and the parallel end direction is the crack. When the angle of the joint is 30°, the direction of crack generation is mainly along the joint, and some cracks are perpendicular to the direction of the end. When the angle of the joint is 45°, the crack direction is mainly along the joint. When the angle of the node is 60°, the direction of crack generation is similar to that of the node at 30°. When the joint angle is 90°, the crack direction is the vertical end direction. The damage process mentioned above is consistent with the damage result of the indoor test.

During the process of rock specimen damage, the tendency of crack development from the edge of the rock specimen to the center of the rock specimen is shown, such as the first splitting tensile damage; some cracks are produced, then increase, and then completely break. The main reason for this phenomenon is that the tensile strength of the slate is much less than the compressive strength. When the stress wave is transmitted to the surface of the rock sample, the splitting tensile damage occurs first. The process of crack formation and development in rock specimens is rapid and difficult to observe in indoor experiments, but any time interval can be observed in numerical simulation.

## Determination of the parameters of the natural joint angle slate HJC constitutive model

### Formula proposed

In this section, based on the results presented in Section "Determination of the HJC constitutive model parameters for joint angles", the influence of the slate seam angle on the parameters of the high-strength HJC constitutive model parameters is analyzed, the fitting relationship between the parameters of HJC constitutive model parameters and slate seam angle is derived, and a numerical simulation method for the dynamic impact of slate at a natural seam angle is proposed.

**Figure 15:**
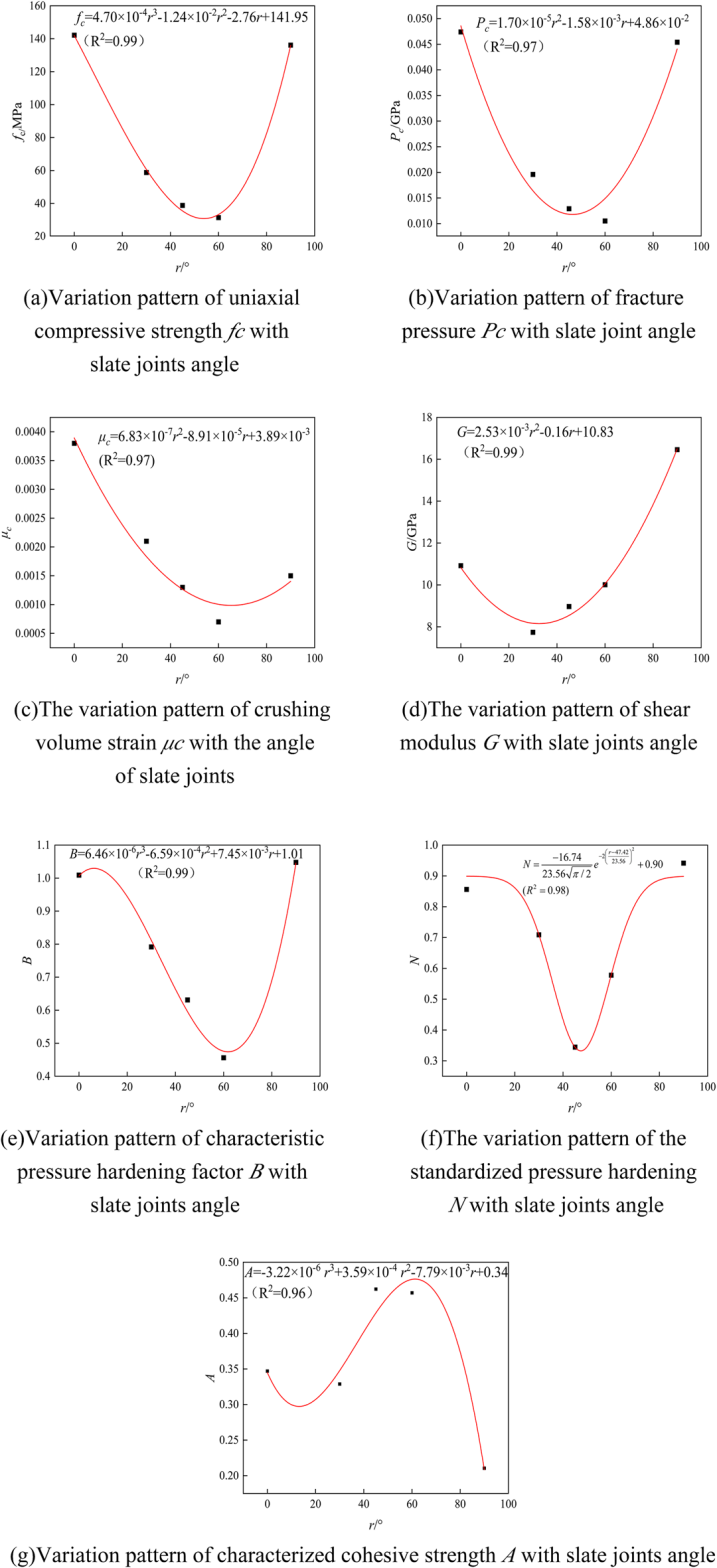
Parameter fitting of HJC constitutive structure model. (**a**) Variation pattern of uniaxial compressive strength *f*_*c*_ with slate joint angle; (**b**) variation pattern of fracture pressure *P*_*c*_ with slate joint angle; (**c**) variation pattern of crushing volume strain *μ*_*c*_ with the slate joint angle; (**d**) variation pattern of shear modulus *G* with slate joint angle; (**e**) variation pattern of characteristic pressure hardening factor *B* with slate joint angle; (**f**) variation pattern of standardized pressure hardening *N* with slate joint angle; (**g**) variation pattern of characterized cohesive strength *A* with slate joint angle.

According to the results in Section "Determination of the HJC constitutive model parameters for joint angles", although density *ρ* is a sensitive parameter, it is an intrinsic feature of the rock and is unaffected by the angle of the node. The remaining parameters are insensitive, so we analyze only parameters *G*, *A*, *B*, *N*, *F*_*c*_, *P*_*c*_, and *μ*_*c*_. The fitting of parameters *G*, *A*, *B*, *N*, *F*_*c*_, *P*_*c*_, and *μ*_*c*_ with the inclination of the slate joint is shown in Fig. 15, and the fitted expressions are shown in Table 5.

**Table 5:** Fitting expressions.

Parameters	Fitting expressions	Goodness of fit
Uniaxial compressive strength *f*_*c*_	$F_{c} = 4.70 \times 10^{ - 4} r^{3} - 1.24 \times 10^{ - 2} r^{2} - 2.76r + 141.95$	0.99
Crushing pressure *P*_*c*_	$P_{c} = 1.70 \times 10^{ - 5} r^{2} - 1.58 \times 10^{ - 3} r + 4.86 \times 10^{ - 2}$	0.97
Crushing volume strain *μ*_*c*_	$\mu_{{\text{c}}} = 6.83 \times 10^{ - 7} r^{2} - 8.91 \times 10^{ - 5} r + 3.89 \times 10^{ - 3}$	0.97
Shear modulus *G*	$G = 2.53 \times 10^{ - 3} r^{2} - 0.16r + 10.83$	0.99
Pressure hardening factor *B*	$B = 6.46 \times 10^{ - 6} r^{3} - 6.59 \times 10^{ - 4} r^{2} + 7.45 \times 10^{ - 3} r + 1.01$	0.99
Standardized pressure hardening index *N*	$N = \frac{ - 16.74}{{23.56\sqrt {\pi /2} }}e^{{ - 2\left( {\frac{r - 47.42}{{23.56}}} \right)^{2} }} + 0.90$	0.98
Characteristic cohesive strength *A*	$A = - 3.22 \times 10^{ - 6} r^{3} + 3.59 \times 10^{ - 4} r^{2} - 7.79 \times 10^{ - 3} r + 0.34$	0.96

As can be seen from Fig. 15a, the uniaxial compressive strength fc decreases first and then increases with the joint angle. When the joint angle *r* = 0°, the damage takes the form of compression damage. When the joint angle *r* < 53.9°, the joint surface is subjected to both positive and shear stress, and when the positive or shear stress of the joint surface exceeds the limit, the rock is damaged. When the joint angle *r* > 53.9°, the shear force on the surface of the node destroys the rock, but the node cuts the rock into rock columns for support, and the uniaxial compressive strength increases gradually.

As can be seen from Fig. 15b, the inclination of the joint increases. As shown in *p*_*c*_ = *f*_*c*_/ 3, the elastic limit hydrostatic pressure pc decreases before increasing, and the pc trend is the same as that of fc.

As can be seen from Fig. 15c, the crushing volume strain *μ*_*c*_ first decreases and then increases with the increase in the joint inclination angle. From *μ*_*c*_ = *P*_*c*_/*K* = *P*_*c*_ (1 − 2*μ*)/*E* and the experimental values, it can be seen that Poisson's ratio *μ* fluctuates less with the variation of the joint inclination angle, and *μ* is the main variation factor of the inelastic ultimate volume strain. From *E* = *σ*/*ε* and the experimental data, the elastic modulus *E* decreases first and then increases as the joint dip angle increases, and the elastic modulus *E* has the same tendency as *p*_*c*_. Therefore, *μ*_*c*_ tends to decrease first and then increase.

As can be seen from Fig. 15d, the shear modulus *G* decreases and then increases as the inclination of the node increases. As can be seen from *G* = *E*/2(1 + *μ*), the elastic modulus *E* is the main variation factor of the shear modulus *G*. For elastic modulus *E* = *σ*/*ε*, small variations in Poisson's ratio *μ* show that epsilon varies less with the joint inclination and a non-major influencing factor. The change in elastic modulus *E* is mainly influenced by sigma, the change in shear modulus *G* is mainly influenced by stress *σ*, and the change in shear modulus *G* is similar to that of *f*_*c*_.

As can be seen from Fig. 15e,f, the standardized pressure hardening coefficient *B* and the standardized pressure hardening index *N* decrease with the joint inclination and then increase again. Standard pressure hardening factor *B* and standard pressure hardening index *N* are parameters that measure the degree of hardening of materials and are associated with equivalent force σ^*^ and standardized hydrostatic pressure P^*^. From the experimental data, it can be seen that σ^*^ and P^*^ tend to decrease first and then increase as the inclination of nodes increases. Therefore, the standard pressure hardening factor *B* and the standard pressure hardening index *N* show a tendency to decrease before rising.

As can be seen from Fig. 15g, the characteristic bond A decreases as the pitch of the joint increases, then increases, then decreases. From *A* = *c*/*fc* (1 + *C*Ln10^–4^), *c* = *σ*_*c*_ (1 − sin*φ*)/2cos*φ*, and the experimental values, the cohesive force *C* is mainly affected by the intercept of the function in the graph of internal friction angle *φ* versus the axial pressure–surrounding pressure relationship. However, the change in cohesive force *c* is small compared to *f*_*c*_, so the characteristic cohesive strength *A* shows a decrease, then an increase, and then a decrease under the synergistic change in cohesive force *c* and *f*_*c*_.

As can be seen from Table 5, we used polynomial functions and Gaussian functions to perform nonlinear fitting of the joint inclination angle with some of the sensitivity parameters of the HJC constitutive model; the R^2^ values of the fitting results are all above 0.95, and the fitting results are good.

### Formula validation

**Figure 16:**
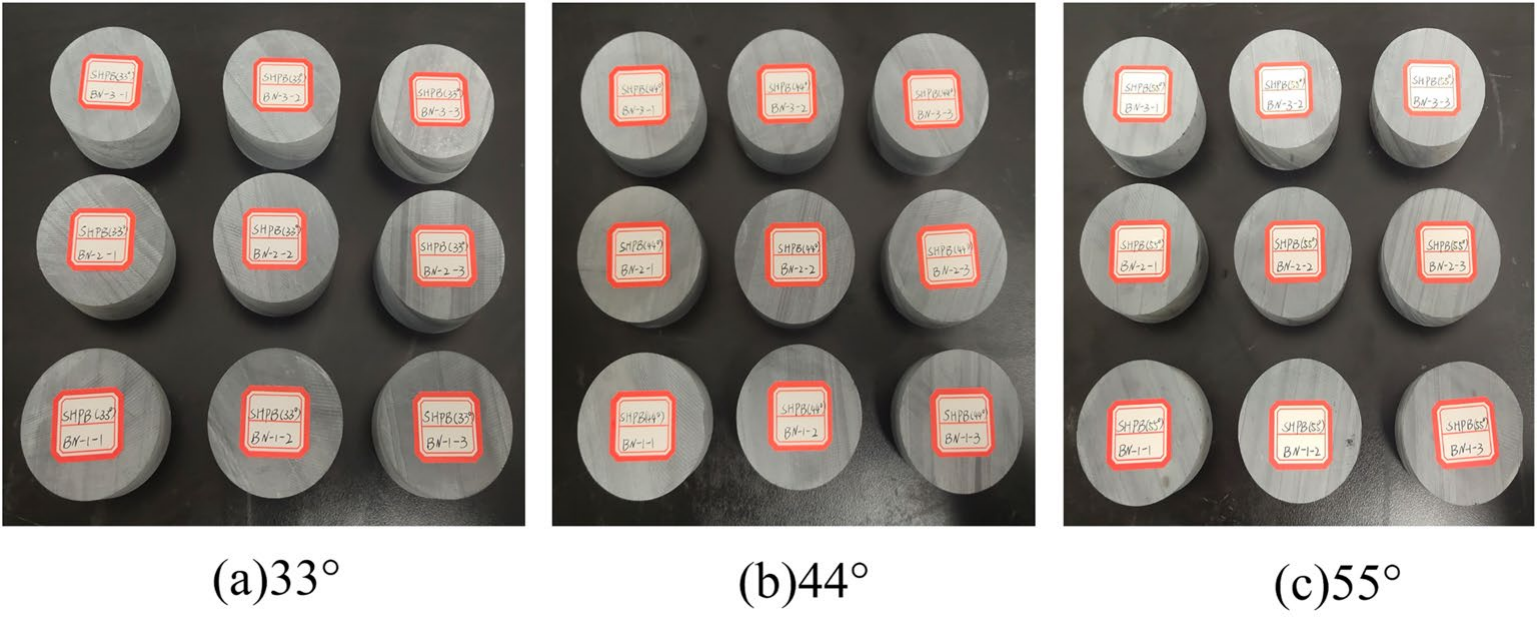
Slate rock sample. (**a**) 33°; (**b**) 44°; (**c**) 55°.

**Figure 17:**
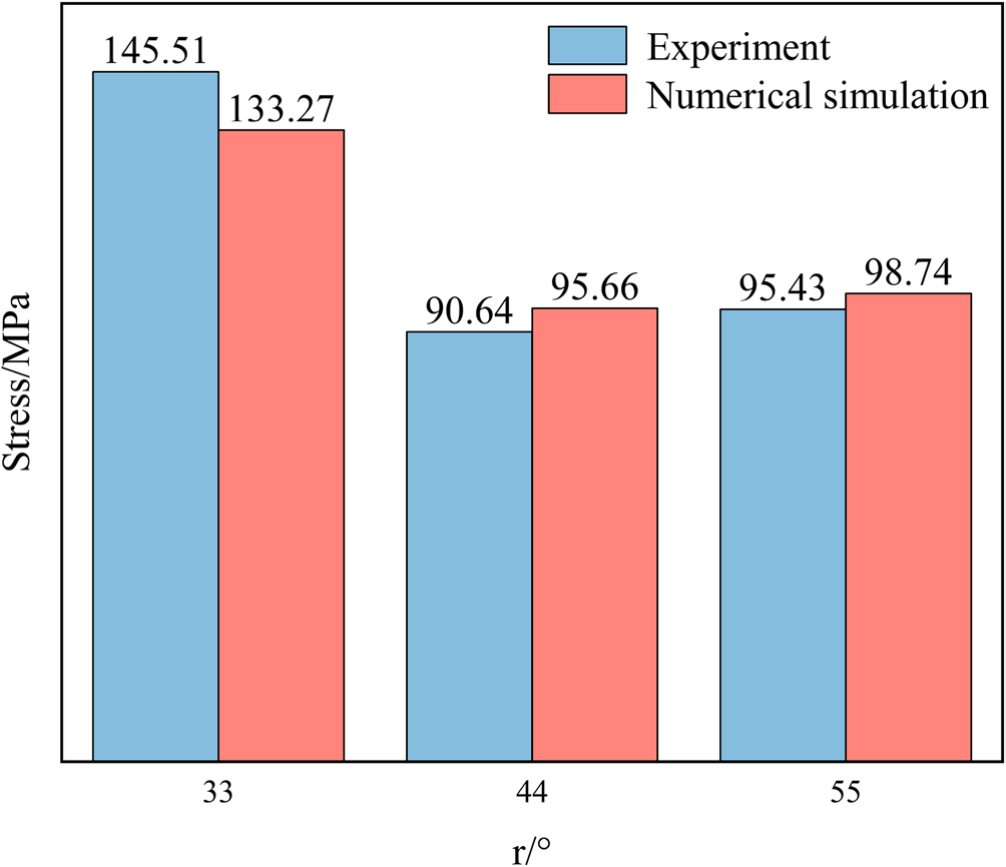
Comparison of experimental and numerical simulation results.

In order to verify the conclusions of the theoretical analysis, core samples were drilled and processed as standard specimens at the Tongzi Tunnel site in Guizhou, China. The peak stresses of three groups of natural contact slate at 33, 44, and 55° were determined through three types of natural contact SHPB impact tests, and the impact pressure was 0.3MPa (see Fig. 16). A comparison of the experimental results with the numerical simulation results is shown in Fig. 17.

As can be seen from Fig. 17, the SHPB numerical simulations of peak stress were performed on slate specimens with nodal angles of 33°, 44°, and 55°, based on the formula presented in Table 5, and were compared to indoor test results with numerical accuracies of 91.59%, 94.75%, and 96.65%, respectively. The numerical simulation results are good, and the proposed formula is applicable.

## Conclusions

1. Sensitivity analysis was conducted on 21 parameters of the HJC constitutive model with the dynamic peak stress of the slate as the objective function. The effect of parameters *ρ*, *G*, *A*, *B*, *N*, *F*_*c*_, *P*_*c*_, and *μ*_*c*_ on the dynamic peak stress of the slate is greater than 10%. Parameters *A* and *B* have the greatest effect on the dynamic peak stress of the slate at over 50%.
2. With respect to the parameters of the HJC constitutive model using special slate joint inclination angles, the numerical simulation accuracy lies within the interval (91.68%, 99.94%), which verifies the accuracy of the parameter determination method.
3. With the increase in the joint angle, the uniaxial compressive strength fc decreases first and then increases. When the joint angle r < 53.9°, the joint surface is simultaneously subjected to positive stress and shear stress. When the positive stress or shear stress on the joint surface exceeds the limit, the rock will be damaged. When the joint angle r > 53.9°, the rock body is cut by the joint to form a rock column to provide support, so the uniaxial compressive strength gradually increases. From *P*_*c*_ = *f*_*c*_/3, the trend in *P*_*c*_ is the same as the trend in *f*_*c*_. The crushing volume strain *μ*_*c*_ is mainly affected by the variation in elastic modulus *E* and *P*_*c*_, and the crushing volume strain *μ*_*c*_ tends to decrease first and then increase. The trend in shear modulus *G* is mainly influenced by *f*_*c*_, and the trend is approximately the same as *f*_*c*_. Standardized pressure hardening factor *B*, standardized pressure hardening index *N*, and equivalent force σ^*^ correlate with standardized hydrostatic pressure P^*^, first decreasing and then increasing with the increasing joint angle. The characteristic cohesive strength *A* decreases, then increases, and then decreases under the synergistic variation of cohesive forces *c* and *f*_*c*_.
4. The HJC constitutive model parameters and the slate joints have an excellent fitting relationship, and the HJC constitutive model parameters of the slate under the natural joints exhibit good agreement between the indoor testing and numerical simulation results, with the fitting accuracy reaching 0.96 ~ 0.99. The results provide a simple and feasible numerical simulation method for slate dynamics analysis.
5. This article is based on the indoor test results of five special joint dip angle layered slate, combined with nonlinear fitting regression method, to determine the HJC constitutive model parameters of natural joint dip angle layered slate. The accuracy of the constitutive parameters was verified through indoor experiments. The error between numerical simulation and indoor test results can be controlled within 10%, and the simulation accuracy is high. The research results provide a high-precision numerical simulation method for similar projects.

## Data Availability

The datasets used during the current study available from the corresponding author on reasonable request.
